# Impact of patient-prosthesis mismatch on 30-day outcomes in young and middle-aged patients undergoing aortic valve replacement

**DOI:** 10.1186/1749-8090-7-46

**Published:** 2012-05-15

**Authors:** Daniel Hernández-Vaquero, Juan C Llosa, Rocío Díaz, Zain Khalpey, Carlos Morales, Rubén Álvarez, Jose López, Francisco Boye

**Affiliations:** 1 Cardiac Surgery Department, Hospital Universitario Central de Asturias, Oviedo, Spain; 2 Cardiac Surgery Department, Brigham and Women’s Hospital, Harvard Medical School, Boston, USA; 3 Cardiac Surgery Department, Children Hospital Boston, Harvard Medical School, Boston, USA

**Keywords:** Patient-prosthesis mismatch, Severe aortic stenosis

## Abstract

**Background:**

The impact of patient-prosthesis mismatch (PPM) on early outcomes in young and middle-aged patients undergoing conventional aortic valve replacement for severe aortic stenosis remains unknown. Our objective was to evaluate the incidence of some degree of PPM and its influence on early mortality and morbidity.

**Methods:**

We analyzed our single center experience in all patients <70 years undergoing first-time isolated aortic valve replacement for severe stenosis in our center from September 2007 to September 2011. PPM was defined as an indexed effective orifice area ≤ 0,85 cm^2^/m^2^. The influence of PPM on early mortality and postoperative complications was studied using propensity score analysis. Follow up at 30 postoperative days was 100% complete.

**Results:**

Of 199 patients studied, 61 (30,7%) had some degree of PPM. PPM was associated with an increased postoperative mortality (OR = 8,71; 95% CI = 1,67–45,29; *p* = 0,04) and major postoperative complications (OR = 2,96; CI = 1,03–8,55; *p* = 0,044). However, no association between PPM and prolonged hospital or ICU stay was demonstrated.

**Conclusions:**

Moderate PPM is a common finding in young and middle-aged patients undergoing surgery for aortic valve replacement due to severe stenosis. In addition, its influence on early outcomes may be relevant.

## Background

Aortic stenosis is the most common valvular heart disease and the third most common cardiovascular disorder after hypertension and coronary artery disease in the developed world [1]. Left ventricular hypertrophy caused by severe aortic stenosis (SAS) is associated with sudden death, congestive heart failure and stroke [2]. Aortic valve replacement (AVR) has been shown to change the natural history of these patients [1] reducing the pressure gradient between the left ventricle and ascending aorta and reversing left ventricular remodeling. However, if some degree of residual aortic stenosis remains after replacement reverse remodeling may be compromised.

Patient-prosthesis mismatch (PPM) after an AVR was first defined by Rahimtoola [3] as the situation in which the effective area of a well-functioning prosthetic valve is less than that of a normal human valve. However, despite the almost 35 years since its original description, the concept and impact of PPM remains highly controversial. Whereas some researchers have reported a lower post-operative survival rate among patients with PPM [4,5], others have not observed these adverse results [6,7].

Given this situation, it has been recently suggested that the impact of PPM on outcomes depends on baseline patient characteristics. Accordingly, some studies [8,9] have revealed that PPM has a significant negative effect on long-term survival of patients < 70 years old but not in the elderly.

Moreover, ventricular performance and hemodynamic status are more vulnerable during the early postoperative period when the increased afterload induced by PPM may be particularly deleterious, resulting in an increased postoperative mortality [2].

However, at our knowledge no previous reports have investigated the incidence and the impact of PPM on mortality and morbidity during the first 30 postoperative days in patients under 70 years after conventional AVR. For this purpose, we studied a consecutive series of that subgroup of patients who underwent isolated first-time AVR for SAS in our center.

## Methods

### Patient population and data collection

Between September 2007 and September 2011, 199 patients > 18 and < 70 years old underwent isolated first-time AVR for SAS with or without some degree of associated aortic regurgitation in the Cardiac Surgery Department of the *Hospital Universitario Central de Asturias* (Oviedo, Spain). To avoid a heterogeneous series, patients with significant coronary artery disease, those with previous percutaneous coronary interventions, or those who required concomitant mitral or tricuspid valve surgery or ascending aorta replacement were excluded.

Coronary angiography and transthoracic echocardiography were performed in all patients within the 12 and 6 months prior to the intervention respectively. Clinical, operative, and outcomes data were prospectively collected and validated. Database was queried retrospectively. All discharged patients were clinically assessed one month after the operation in our outpatient clinic. Follow-up at 30 postoperative days was 100% complete. The study was approved by the regional ethics committee.

### Definition of PPM and study end-points

PPM was defined as the indexed effective orifice area (IEOA) ≤ 0,85 cm^2^/m^2^ and was considered severe when the IOEA was ≤ 0,65 cm^2^/m^2^[10,11]. To calculate the IEOA we divided between the *in vivo* EOA measurements for each prosthesis and the body surface area (BSA) which was calculated using the Dubois formula [6]. We used the data published for the *in vivo* EOA values [12-17] (Table 1) due to its ability to predict the postoperative gradients [4,18,19], whereas *ex-vivo* measurements reported by manufacturers overestimate the true prosthetic valve EOA [13,18], and consequently, result in a low sensitivity for the prediction of PPM [11].

**Table 1:** ***In vivo*effective orifice area values corresponding to each valve**

**Prosthetic valve**	**19 mm**	**21 mm**	**23 mm**	**25 mm**	**Reference**
*Mechanical*					
St Jude M Regent	1,6 (22)	2 (42)	2,2 (26)	2,5 (17)	[12]
Carbomedics	1 (1)	1,5 (9)	1,6 (22)	2 (11)	[13]
Carbomedics Top Hat	1,1 (4)	1,2 (4)	1,4 (16)	1,6 (0)	[14]
MCRI On-X	1,5 (2)	1,7 (0)	2 (1)	2,4 (0)	[15]
*Bioprosthesis*					
Mitroflow	1,2 (3)	1,4 (10)	1,6 (7)	(0)	[16]
Labcor	(0)	1,1 (1)	1,4 (0)	1,5 (0)	[17]

In parentheses, the number of patients with that valve in our series.

We analyzed the influence of PPM on 30 day mortality and postoperative complications. For statistical power enhancement, postoperative complications were pooled into major complications: postoperative acute myocardial infarction (AMI), stroke, reoperation for bleeding and new need for balloon counterpulsation and minor complications: pericardial drainage, new need for permanent pacemaker, pneumonia, persistent atrial fibrillation (AF) and late extubation. The influence of PPM on intensive care unit (ICU) and total hospital stay was separately analyzed.

### Echocardiographic assessment

All the patients underwent a complete M-mode, 2-dimensional, and Doppler transthoracic echocardiography before surgery. Left ventricular ejection fraction was calculated using Simpson’s formula from biplane apical four and two chambers views. The severity of aortic stenosis was graded by integration of Doppler methods, continuity equation and planimetry and the degree of aortic regurgitation was determined primarily by the width of the regurgitant jet determined by color Doppler and then calculating the regurgitant orifice area and characterizing the reversed flows in descending aorta.

### Surgical technique

All patients were approached through a standard full median sternotomy, under cardiopulmonary bypass with moderate hypothermia. Myocardial protection was accomplished by antegrade and retrograde cristaloid cardioplegia with Celsior® solution (Genzyme, United States). The largest suitable prosthesis was always implanted in supra-annular position using the specific manufacturer’s devices. Valvular prostheses were implanted with mattress sutures with teflon pledgets. The final decision as to the type of prosthesis to be implanted was made by the surgeon at the time of surgery taking into account the preoperative characteristics and the intraoperative findings.

### Statistical analysis

Shapiro-Wilk test was used to verify the normality of quantitative variables. Continuous variables were expressed as mean ± standard deviation or median (interquartile range), as appropiate. Categorical variables were expressed as absolute number (percentage). Comparisons of proportions were performed using the *χ*^2^ test or Fisher’s exact test, as appropriate. Group comparison for continuous variables was tested with the Student’s unpaired *T* test in case of normal distribution or Mann–Whitney *U* test otherwise.

To minimize potential selection bias typical of observational reports, a propensity score (PS) analysis was undertaken. This is the probability that PPM occurs in a patient given his or her baseline and surgical characteristics. To calculate PS we created a non-parsimonious logistic regression model where PPM acted as a dependent variable and, as predictors, all the variables that differed according to the PPM in the univariate analysis (Tables 2 and 3) and those that, although did not differ, were considered clinically relevant. We verified the supposition of linearity for the continuous variables and the lack of colinearity between the predictors. The discrimination and calibration of the model were evaluated by area under the receiver operating characteristic curve and Hosmer-Lemeshow test for goodness of fit.

**Table 2:** Preoperative patient characteristics

	**Without PPM**	**With PPM**	**p**
Age	61 (56–66)	64 (57–68)	0,046
Women	49 (35,5%)	18 (29,5%)	0,41
HT	77 (55,8%)	34 (55,7%)	0,99
DM	22 (15,9%)	15 (25%)	0,13
Hypercholesterolemia	70 (50,7%)	27 (44,3%)	0,4
BSA	1,82 ± 0,2	1,91 ± 0,16	0,002
BMI	28,84 ± 4,29	30,07 ± 4,27	0,086
Peripheral arterial disease	13 (9,4%)	11 (18%)	0,085
CPD	20 (14,5%)	11 (18%)	0,52
Previous stroke	4 (2,9%)	4 (6,6%)	0,22
Previous neurological dysfunction	4 (2,9%)	0 (0%)	0,18
Preoperative creatinine concentration	0,94 (0,8–1,09)	1 (0,86–1,17)	0,043
Previous AF	18 (13%)	7 (11,5%)	0,76
Previous AMI	1 (0,7%)	1 (1,6%)	0,55
LVD	16 (11,6%)	7 (11,5%)	0,98
SPHT	14 (10,1%)	9 (14,8%)	0,35
Peak transaortic pressure gradient	81 (70–91)	81 (71–93)	0,93
Associated aortic regurgitation	27 (19,6%)	16 (26,2%)	0,29
Emergency surgery	4 (2,9%)	1 (1,6%)	0,61
Logistic EuroSCORE	2,8 (2,1–4,6)	2,9 (2,1–5,1)	0,51
Previous pacemaker implantation	0 (0%)	2 (3,3%)	0,033
NYHA functional class III-IV	67 (48,6%)	30 (49,2%)	0,93
*Etiology*			
Degenerative disease	90 (65,2%)	41 (67,2%)	0,78
Congenital disease	37 (26,8%)	13 (21,3%)	0,41
Rheumatic disease	11 (8%)	7 (11,5%)	0,43

HT: HYPERTENSION; DM: DIABETES MELLITUS; BSA: BODY SURFACE AREA; BMI: BODY MASS INDEX; COPD: CHRONIC PULMONARY DISEASE; AF: ATRIAL FIBRILLATION; AMI; ACUTE MYOCARDIAL INFARCTATION; LVD: LEFT VENTRICULAR DYSFUNCTION; SPHT; SEVERE PULMONAR HYPERTENSION; NYHA: NEW YORK HEART ASSOCIATION.

**Table 3:** Characteristics of the surgical procedure

	**Without PPM**	**With PPM**	**p**
Previous balloon counterpulsation	0 (0%)	1 (1,6%)	0,13
CPB time	68 (56–80)	76 (67–101)	0,004
Aortic cross-clamp time	54 (46–65)	62 (48–71)	0,032
Bioprosthesis	6 (4,3%)	14 (23%)	<0,001

CPB: CARDIOPULMONAR BYPASS.

Odds ratios (ORs) were calculated by a logistic regression model for in-hospital outcomes with PPM as the exposure variable and PS as the covariate. All analyses were stratified by main surgeon and year of surgery. The *Enter-method* was employed in all regression models and a two-sided *p* value < 0,05 was considered statistically significant.

### Definitions

Severe aortic stenosis: aortic valve area of less than 1 cm^2^, mean gradient greater than 40 mmHg or jet velocity greater than 4 m/s measured by preoperative echocardiography.

Left ventricular dysfunction (LVD): left ventricular ejection fraction < 50%.

Severe pulmonary hypertension (SPHT): systolic pulmonary artery pressure > 50 mmHg.

Coronary artery disease: > 50% reduction in vessel diameter in at least one angiographic plane.

Associated aortic regurgitation: concomitant grade III or IV aortic regurgitation by transthoracic echocardiography.

Emergency surgery: the operation that is required within 24 hours since onset of symptoms due to unstable critical status or life-threatening situation.

Chronic pulmonary disease: long term use of bronchodilators or steroids for lung disease.

Peripheral arterial disease: carotid stenosis > 50%, claudication or previous or planned intervention on the abdominal aorta, limb arteries or carotids.

Postoperative period: that conducted during hospital stay or 30 days after surgery if previously discharged.

Postoperative AMI: troponin T level ≥ 1 ng/ml associated with compatible electrocardiographic (usually new left bundle branch block or new Q waves) and echocardiographic findings.

Postoperative stroke: clinically compatible neurological event that persists for at least 24 hours.

Persistent postoperative AF: atrial fibrillation at discharge in patients with preoperative sinus rhythm.

Late extubation: carried out after the first 24 hours post-surgery.

Prolonged ICU and total hospital stay: 75 percentile of our series was selected (more than 3 days for ICU and more than 11 days for total hospital stay).

## Results

### Patient characteristics and surgical data

**Figure 1:**
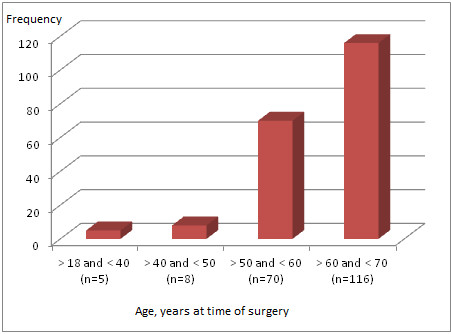
Age distribution at the time of surgery.

During the study period, 199 patients met the inclusion criteria. Sixty-seven (33,7%) were women and the median age was 62 (57–67) years old (Figure 1).

Sixty-one (30,7%) patients had some degree of PPM and their IEOA was 0,76 (0,71–0,81) versus 1,08 (0,95–1,19) in the patients with no PPM. Only 6 (3%) patients had severe PPM. As depicted in Table 2, patients with PPM were older and had higher body surface area, higher preoperative creatinine levels and greater proportion of previous pacemaker. In addition, patients with PPM had longer cardiopulmonary bypass and aortic cross-clamp times and greater percentage of tissue valves implanted (Table 3).

### Postoperative mortality

Five patients (8,2%) with and one (0,7%) without PPM died during the postoperative period (*p* = 0,004). All deaths were cardiac-related (three patients died due to myocardial infarction, two patients due to low cardiac output syndrome with cardiogenic shock and one due to sudden death in hospital ward). No patient died between discharge and postoperative day 30.

The following variables were included for PS model construction: age, gender, hypertension, BSA, emergency surgery, previous pacemaker, preoperative creatinine concentration, peak transaortic pressure gradient, LVD, associated aortic regurgitation, III-IV *New York Heart Association* (NYHA) functional class, cardiopulmonary bypass time, aortic cross-clamp time, type of prosthesis, main surgeon and year of surgery. The estimated PS showed good discrimination power (C statistic = 0,78; 95% CI = 0,71–0,85) and calibration characteristics (*χ*^2^ = 4,95; *p* = 0,76) After adjusting for PS, PPM was a strong predictor of postoperative mortality (OR = 8,71; 95% CI = 1,67–45,29; *p* = 0,04).

### Postoperative complications and stay

Table 4 shows postoperative complications of patients with and without PPM. When compared with patients with no PPM, patients with PPM had more percentage of postoperative stroke and new need for balloon counterpulsation. Total hospital stay was 9 (7–11) days for patients with PPM and 9 (7–11) days for patients without PPM (*p* = 0,91). ICU stay was 2 (1–3) for patients with PPM and 2 (1–3) for those with no PPM (*p* = 0,73).

**Table 4:** Postoperative complications

	**Without PPM**	**With PPM**	**p**
*Major complications*			
Postoperative AMI	5 (3,6%)	5 (8,2%)	0,17
Postoperative stroke	0 (0%)	3 (4,9%)	0,009
Reintervention due to bleeding	4 (2,9%)	3 (4,9%)	0,47
New need for balloon counterpulsation	4 (2,9%)	9 (14,8%)	0,002
*Minor complications*			
Pericardial drainage	2 (1,4%)	3 (4,9%)	0,15
Persistent AF	6 (5,1%)	3 (6%)	0,82
Pneumonia	5 (3,6%)	0 (0%)	0,13
New need for permanent pacemaker	4 (2,9%)	1 (1,6%)	0,6
Late extubation	12 (8,8%)	9 (15%)	0,19

AMI: ACUTE MYOCARDIAL INFARCTION; AF: ATRIAL FIBRILLATION.

After adjusting for PS, PPM was an independent predictor for major postoperative complications (OR = 2,96; CI = 1,03–8,55; *p* = 0,044) but not for minor complications (OR = 0,61; CI = 0,23–1,6; *p* = 0,31). In addition, PPM did not show influence on prolonged ICU stay (OR = 0,65; CI = 0,32–1,34; *p* = 0,24) or prolonged total hospital stay (OR = 0,91; CI = 0,43–1,91; *p* = 0,81).

## Discussion

The incidence of PPM in our single-centre cohort of young and middle-aged patients who underwent AVR for SAS was 30,7%. Although this is in line with the rates reported on some studies [4,9], these results are highly variable in the literature being present in 19–70% of patients [2]. In addition, the influence of PPM on the outcomes of patients undergoing AVR is unknown and remains controversial. Several reasons could be argued to explain these diverging results.

First, different methods have been used for PPM calculation. Recently, *in vivo* EOA measurements are increasingly being selected for this purpose, but some reference authors [5,7] still use *ex vivo* manufacturer reported EOA values, with the limitations derived from this fact [11]. Secondly, contemporary standard AVR offers excellent early and late clinical outcomes with a low incidence of adverse events which makes it difficult to see differences. And finally, the heterogeneity of the population previously studied, so most previous reports have studied patients with other valvular diseases, ascending aortic aneurysms or concomitant bypass grafting.

It has recently been suggested that the impact of PPM could be influenced by patient baseline conditions. So, some researchers are focusing on identifying those patients who are more vulnerable to the clinical consequences of PPM. Mohthy et al. [8] and Moon et al. [9] have found a significant negative effect of PPM on late survival in patients < 70 and < 60 years old respectively. However, at our knowledge no previous reports have studied in young patients the impact of PPM on mortality and morbidity during the early postoperative course which is probably the most vulnerable period for the left ventricle [2].

### Impact of PPM on early mortality

Although it is of utmost importance to assess the impact of PPM on early mortality after AVR, findings across several studies show conflicting results. This is often due to the wide heterogeneity between studies. As aforementioned, there are at least 2 different mismatch entities (severe and moderate PPM), several parameters used to calculate PPM (*in vivo* or *ex vivo* EOA values) and several cut-off points to consider its existence.

Bridges et al. [20] published the largest study on PPM, which analyzed data acquired from a total of 42.310 patients undergoing isolated AVR. Small EOA were reported to be associated with increased operative mortality, but among patients receiving the same prosthesis model and size, those patients with a larger BSA had better outcomes. It was speculated that the impact of PPM on short term mortality may be less important than several unmeasured confounding variables, including the BSA.

Urso et al. [6] have recently reviewed the concept of mismatch as a risk factor for early and mid-term mortality after AVR. These authors found no association between PPM and early outcomes. However, it should be noted that the patient population was elderly with a median age of 72. Other authors as Blais et al. [4] or Rao et al. [5] studied large series of patients undergoing AVR and found that PPM was a significant predictor of early mortality.

Our findings, in a consecutive series of 199 young and middle-aged patients (median age of 62 years) with a low early mortality rate, suggest that even moderate PPM, negatively impact on early postoperative survival after isolated AVR for SAS.

### Impact of PPM on postoperative complications

Although mortality is the most important postoperative adverse outcome, lower morbidity rates are essential for adequate patient recovery and quality of life. In fact, the presence of excellent perioperative outcomes after AVR, factors possibly affecting the longer term functionality gain more and more importance. Our study showed that in young patients undergoing AVR, PPM is associated with major complications, mostly at the expense of postoperative stroke and new need for balloon counterpulsation. In this sense, Nozohoor et al. [7] reported that PPM was a predictor of postoperative neurological events in general population undergoing surgery. The authors argue possible reasons for this finding, such as a more cumbersome surgical procedure in those patients with small aortic annuli and extensive calcification who are prone to PPM. In addition, the increased post-prosthetic turbulence may induce rupture of calcified plaques with subsequent embolization. However, Yap et al. [21] did not find an association between PPM and stroke, prolonged ventilation and prolonged ICU or hospital stay.

Our study failed to demonstrate significant association between PPM and prolonged hospital stay. An underpowered analysis or the absence of differences in minor complications could have played a key role in this finding. Moreover, further studies with larger number of patients will be necessary to define which specific complications are related to PPM.

### Clinical implications

The major finding of this study is that PPM is an important and independent risk factor for short-term mortality in young and middle-aged patients undergoing AVR. The practical implications of these findings are important given that PPM is not a rare occurrence with a prevalence in the literature between 19 and 70% [2,19,22].

Furthermore, as opposed to other predictors of short-term mortality, moderate-severe PPM can be largely avoided with the use of a proper preventive strategy at the time of operation [19,22,23].

### Limitations

Several limitations should be noted. This is a single-center study and results extrapolation must be cautiously considered. In addition, this study is retrospective and has limitations inherent to its nature. Moreover, the sample size is relatively small with few adverse clinical events and therefore, a random change in one of them could possibly modify the final results.

## Conclusions

Although severe PPM is rare, moderate mismatch is a common finding in the young and middle-aged patient undergoing AVR for SAS. Our results suggest that, even when moderate, the presence of PPM confers a worse short-term prognosis, both in terms of perioperative complications and mortality. Consequently, preventive surgical strategies are strongly recommended for this patient population.

## Abbreviation

SAS, Severe aortic stenosis; AVR, Aortic valve replacement; PPM, Patient-prosthesis mismatch; I/EOA, Indexed/Effective orifice area; BSA, Body surface area; PS, Propensity score.

## Competing interest

The authors declare that they have no competing interest.

## Authors’ contribution

DH and JCL acted in conception and design. DH, RD, FB, and ZK acted in manuscript writing. DH, and RD acted in acquisition of data, analysis and interpretation. JCL, CM, RA, JL and FB acted in revision of the article providing important intellectual concepts. All authors read and approved the final manuscript.
